# Identification of stable reference genes and differential miRNA expression in Sri Lankan type 2 diabetes mellitus patients: a cross-sectional study

**DOI:** 10.3389/fendo.2025.1554827

**Published:** 2025-06-12

**Authors:** Palihaderu Arachchige Dineth Supasan Palihaderu, Balapuwaduge Isuru Layan Madusanka Mendis, Jayasekara Mudiyanselage Krishanthi Jayarukshi Kumari Premarathne, Wajjakkara Kankanamlage Ruwin Dias, Swee Keong Yeap, Wan Yong Ho, Arosha Sampath Dissanayake, Iyanthimala Harshini Rajapakse, Panduka Karunanayake, Upul Senarath, Dilan Amila Satharasinghe

**Affiliations:** ^1^ Department of Basic Veterinary Sciences, Faculty of Veterinary Medicine and Animal Science, University of Peradeniya, Peradeniya, Sri Lanka; ^2^ Department of Livestock and Avian Sciences, Faculty of Livestock, Fisheries, and Nutrition, Wayamba University of Sri Lanka, Makandura, Gonawila, Sri Lanka; ^3^ Department of North Indian Music, Faculty of Music, University of the Visual and Performing Arts, Colombo, Sri Lanka; ^4^ China-ASEAN College of Marine Sciences, Xiamen University Malaysia, Sepang, Selangor, Malaysia; ^5^ Faculty of Science and Engineering, University of Nottingham Malaysia, Semenyih, Malaysia; ^6^ Department of Clinical Medicine, Faculty of Medicine, University of Ruhuna, Galle, Sri Lanka; ^7^ Department of Psychiatry, Faculty of Medicine, University of Ruhuna, Galle, Sri Lanka; ^8^ Department of Clinical Medicine, Faculty of Medicine, University of Colombo, Colombo, Sri Lanka; ^9^ Department of Community Medicine, Faculty of Medicine, University of Colombo, Colombo, Sri Lanka

**Keywords:** type 2 diabetes mellitus, hsa-miR-425-5p, hsa-miR-22-5p, hsa-miR-29a-3p, hsa-miR-375-3p, microRNA expression, reference, stability

## Abstract

**Introduction:**

Type 2 Diabetes mellitus is a major global health concern. MicroRNA plays an important role in regulating pancreatic beta cells as well as peripheral insulin signaling. This study aimed to identify reference microRNA s in type 2 Diabetes mellitus plasma and validate two target microRNAs among a Sri Lankan population with type 2 diabetes mellitus.

**Methods:**

This is a cross-sectional experiment. A total of fifty-three (N = 53) non-hemolyzed plasma samples from individuals with type 2 diabetes mellitus were selected to evaluate stability, in comparison to thirty-eight (N = 38) normoglycemic non-hemolyzed plasma samples. Initially, the stability of four candidate reference microRNAs (hsa-miR-16-5p, hsa-miR-425-5p, hsa-miR-191-5p, and hsa-miR-22-5p) was assessed. Stability was analyzed using the geNorm and BestKeeper algorithms. The relative expression changes of hsa-miR-29a-3p and hsa-miR-375-3p in the plasma of the same samples were evaluated using the validated reference microRNAs. The selected regulatory microRNAs were directly linked with type 2 diabetes mellitus pathogenesis and proved to be upregulated in type 2 diabetes mellitus plasma and serum.

**Results and discussion:**

The expressions of miR-16-5p and miR-191-5p were not stable between the two groups, miR-22-5p and miR-425-5p levels were found to be stable. A significant upregulation of hsa-miR-29a-3p and hsa-miR-375-3p was observed in type 2 diabetes mellitus patients compared to normoglycemic individuals (p ≤ 0.05). This was the first study to claim hsa-miR-425-5p and hsa-miR-22-5p as stably expressed reference microRNAs in type 2 diabetes mellitus patients. Sri Lankan type 2 diabetic patients also had increased hsa-miR-29a-3p and hsa-miR-375-3p levels. However, large and well-matched sample studies were suggested to ensure that these microRNAs can be used as type 2 diabetes diagnostic markers in Sri Lanka.

## Introduction

Diabetes mellitus (DM) has become a major concern and is recognized as one of the fastest-growing challenges of the 21st century (1). As one of the major DM types, type 2 DM has accomplished the utmost concern in the past few decades due to its increased worldwide emergence. The burden of type 2 DM in the world is being increased at an exponential rate in almost every region in the world (1, 2). Without exception, the prevalence of type 2 DM is rapidly increasing in Sri Lanka.– A recent meta-analysis based on prevalence data from 1990 to 2021 revealed that the prevalence of type 2 DM among the general Sri Lankan population has tripled within two decades (3).

MicroRNAs (miRNAs) are one of the key modulators that act post-transcriptionally on messenger RNA (mRNAs) ultimately regulating the subsequent protein translation and thereby the gene expression (4, 5). These small nc-RNA molecules play a role in the etiology and regulation of DM. In type 2 DM, the ablation of beta-cell survival and function progressed due to impaired glucose homeostasis (2, 6, 7). MiRNA expression patterns in the pancreatic beta-cells are different in type 2 DM patients compared to non-disease individuals and were shown regulatory role in beta-cell survival and function. Insulin’s effects on peripheral tissues, like regulating blood glucose, rely on the insulin signaling (IS) pathway. Key molecules in this pathway, like Insulin signaling receptor (INSR) and glucose transporter 4 (GLUT4) (6), are controlled by miRNAs. Dysregulation of these proteins caused by specific miRNAs can lead to insulin resistance. Furthermore, atypical expression of extracellular miRNAs has been demonstrated in type 2 DM (8). Due to their differential expressions and stability, extracellular miRNAs were often studied for their applicability as non-invasive biological markers for type 2 DM diagnostics, disease stratification, and prognostic determination (9).

Accurately quantifying miRNA expression changes in the extracellular environment relies heavily on the selection of stable reference genes (RG) or reference miRNAs (RMs) whose expression remains constant amidst the disease processes (10, 11). These genes/miRNAs act as internal controls, allowing researchers to normalize the expression of regulatory gene/miRNAs of interest and accurately compare expression levels across different samples (11–13). While several templates have been employed as reference controls in past type 2 DM-related miRNA expression studies, their suitability and stability within the specific context of type 2 DM plasma remain to be thoroughly evaluated (14, 15). However, no single “perfect” RM exists for normalizing circulating miRNAs in all situations. This is because the expression of these miRNAs can change depending on the disease of interest or condition being studied (11, 16). Therefore, researchers need to carefully choose and validate RG/RMs specific to their experiment (study population, sample type, and disease) (11). Prior validation of RMs ensures reliable results when measuring miRNA expressions using RT-qPCR.

In this study, we focused on two objectives. Initially, we aim to assess the potential validation of four candidate RMs in type 2 DM patients. Four miRNAs were selected for this study as the candidate RMs. Those included hsa-miR-16-5p, hsa-miR-22a-5p, hsa-miR-191-5p, and hsa-miR-425-5p. As the second objective, we aimed to determine if we could replicate previous observations of hsa-miR-29a-3p and hsa-miR-375-3p using our newly validated RMs in Sri Lankan type 2 DM patients compared to normoglycemic individuals. To the best of our knowledge, no studies published on the expression levels of miRNAs in the Sri Lankan type 2 DM population making this the first study of this caliber. Though the existing miRNA studies have focused on other diseases, such an approach remains unexplored in Sri Lanka (17, 18). By examining the expression levels of these miRNAs in Sri Lankan individuals with type 2 diabetes, we tried to explore the potential of using them as diagnostic markers for the type 2 DM condition in the Sri Lankan population.

## Methodology

### Ethics approval

The study protocol was reviewed and approved by the ethics committee, Faculty of Medicine, University of Colombo (EC-20-010), and the ethics committee of the National Hospital of Sri Lanka.

### Recruitment of type 2 DM patients and individuals with normoglycemia

Potential type 2 DM patients were recruited from the general medicine ward of the National Hospital of Sri Lanka. Individuals with normoglycemia were recruited from the community from the 1st of March 2022 to the 30th of November 2022. Both type 2 DM patients and normoglycemic individuals were provided with the necessary information and obtained their written consent to participate in the study. This was done initially, either when the subjects attended their clinics or any other place that was convenient for the candidates by trained research assistants. Individuals who consented were initially screened by a trained medical officer. Type 2 DM patients suffering from chronic infections, hepatic and other endocrine metabolic diseases, recent traumas, cancers, hypertension, and macro and microvascular complications were initially screened and excluded. The medical officer evaluated and excluded the individuals who were suspected to be normoglycemic for possible malignant tumors, cardiovascular diseases, or other non-diabetic chronic diseases.

**Figure 1:**
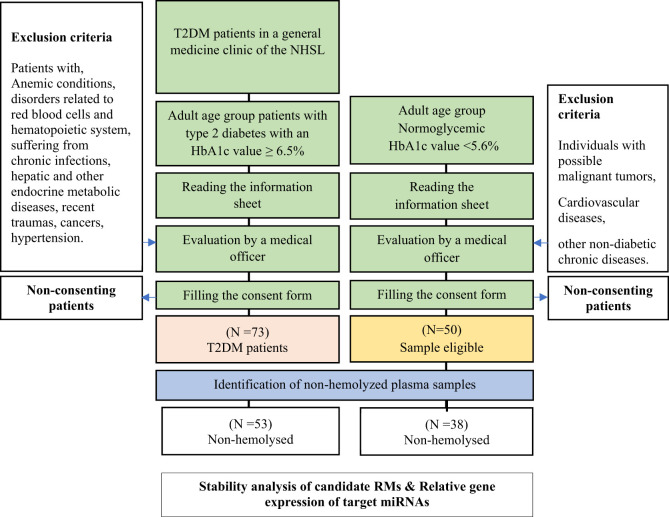
Sample selection and experimental design.

When recruiting normoglycemic individuals, a convenient sampling method was followed. The caretakers, close relatives, or spouses who accompanied type 2 DM patients to the clinic were targeted. The medical officer evaluated and excluded the individuals who were suspected to be normoglycemic for possible malignant tumors, cardiovascular diseases, or other non-diabetic chronic diseases. However, the participants’ candidacy for the selected group was pre-confirmed by an initial Glycated hemoglobin 1c (HbA1c) test. A patient was considered a type 2 DM patient if the HbA1c level was ≥ 6.5% while the subjects with an HbA1c level < 5.6% were considered normoglycemic based on WHO and ADA guidelines (19, 20). HbA1c level, age, and gender were collected as demographics in both groups (**Figure 1**).

### Blood sample collection and plasma separation

Venous blood was drawn and collected in the EDTA-coated tubes (Becton-Dickinson, Franklin Lakes, New Jersey, USA) and stored at 4 °C (21). Venepunctures were conducted by nursing officers. Venous blood was taken into 5.0 mL EDTA-containing tubes. For the HbA1c analysis, 2 mL was used, and for the miRNA analysis, 3 mL was taken. The blood samples for the HbA1c test were delivered to a nearby accredited third-party laboratory. The fraction collected for miRNA analysis was transported at 4 °C to the molecular laboratory within 24 hours (21). Blood was drawn from the type 2 DM patients in the clinic in a sterile environment. Similarly, a sterile environment was maintained when collecting blood from normoglycemic individuals.

The HbA1c was assessed by an ISO 15189 accredited third-party laboratory using the Variant II Turbo Hemoglobin testing system (Bio-Rad, USA). The laboratory used a system that was certified with the National Glycohemoglobin Program (NGSP) and traceable to the International Federation of Clinical Chemistry (IFCC) reference method.

For the miRNA analysis, plasma was separated from normoglycemic individuals and type 2 DM patients following the manufacturer’s protocol (miRNeasy Serum/Plasma Advanced Kit, (QIAGEN, Germany). The blood samples were centrifuged for 10 min at 1900×g (3000 rpm) at 4°C within 24 hours from the blood sample collection. The upper plasma phases were transferred to new micro-centrifuge tubes. To remove the additional cellular nucleic acids attached to the cell debris, the plasma samples were re-centrifuged for 15 min at 3000×g (6200 rpm) at 4°C. The supernatant was transferred to microcentrifuge tubes and immediately stored in 1 mL aliquots at −80 °C until analysis. The frozen plasma was thawed at room temperature (25 °C) before being used for analysis.

### Hemolysis assessment

The levels of hemolysis were measured by spectral analysis using the Nanodrop 2000 spectrophotometer (ThermoFisher Scientific, USA). The absorbance values of each plasma at 414 nm wavelength were measured (22).

### RNA isolation and quality evaluation

Total RNA was from the plasma using miRNeasy Serum/Plasma Advanced Kit (QIAGEN, Germany). The initial plasma volume was 300 µL. Spike–in controls were used to evaluate the extraction and complementary DNA (cDNA) synthesis steps (miRNeasy Serum/Plasma Spike-In Control, Qiagen, Germany). Total RNA was extracted from serum samples stored at -80°C. Samples were thawed at room temperature and mixed with a Spike-In extraction solution within an RPL buffer. Plasma was then added, followed by additional buffers and mixing steps. After centrifugation, the supernatant was transferred and mixed with isopropanol before being loaded onto an RNeasy UCP MinElute column. Sequential washing steps with buffers (RWT and, RPE) and ethanol eliminated impurities. Finally, RNA was eluted with RNase-free water and collected for downstream reactions. Even though RNA stabilizers were not used (23) to prevent RNA degradation, precautions were taken to minimize the RNA loss. Immediately after the isolation, RNA was stored at -80°C.

### cDNA synthesis and quantitative real-time PCR analysis

The total RNA concentration and the RNA integrity were measured using the Nanodrop 2000 spectrophotometer (ThermoFisher Scientific, USA). For miRNA expression analysis, 20ng of the extracted RNA was reverse transcribed using miRCURY LNA RT Kit (QIAGEN, Hildon, Germany) according to the given protocol. The mixture contained 2 µL 5×miRCURY RT Reaction Buffer, 1 µL 10× miRCURY RT Enzyme Mix, RNA template, and RNAse free water. The volume corresponding to 20ng of RNA template was added and topped up the volume to 10 µL using the RNAse-free water.

Then, the reverse transcription was conducted using the CFX96 Touch TM Real-time PCR detection system (Bio-Rad, USA) with a parameter of 42 °C for 60 min, followed by 95 °C for 5 min and 4 °C cooling.

The prepared cDNA was diluted (2-fold) with nuclease-free water before proceeding. The expression was screened using the miRCURY SYBR Green PCR Kit (QIAGEN, Germany). In-house designed and validated primers were used for the analysis of miRNA expression in the CFX96 Touch TM Real-time PCR detection system (Bio-Rad, USA).

The PCR amplification was performed following, a reaction cycle of 95°C for 2 minutes followed by denaturation at 95°C for 10 sec and annealing and extension at 56°C for 60 sec. Melt curve analysis was performed to check the specificity of the amplification products. Meld curves were run from 60°C to 95°C using the CFX96 Touch TM Real-time PCR detection system (Bio-Rad, USA).

### Selection of reference and target microRNAs

The hsa-miR-16-5p, hsa-miR-425-5p, hsa-miR-195-5p, and hsa-miR-22-5p were selected as RMs of interest primarily because they were discovered to be endogenous RMs in the plasma and serum of individuals with diverse diseases. In the human blood plasma, serum, and other organs of patients suffering from various ailments, these selected miRNAs were found to be quite stable, and even previous studies have utilized these miRNAs as reference controls including type 2 DM (S**upplementary Table 1**). Furthermore, according to our prior research survey (from year 2019 -2020), though previous research has shown that these miRNAs have regulatory functions in other diseases, no direct association has been identified between their plasma expressions and type 2 DM.

**Figure 2:**
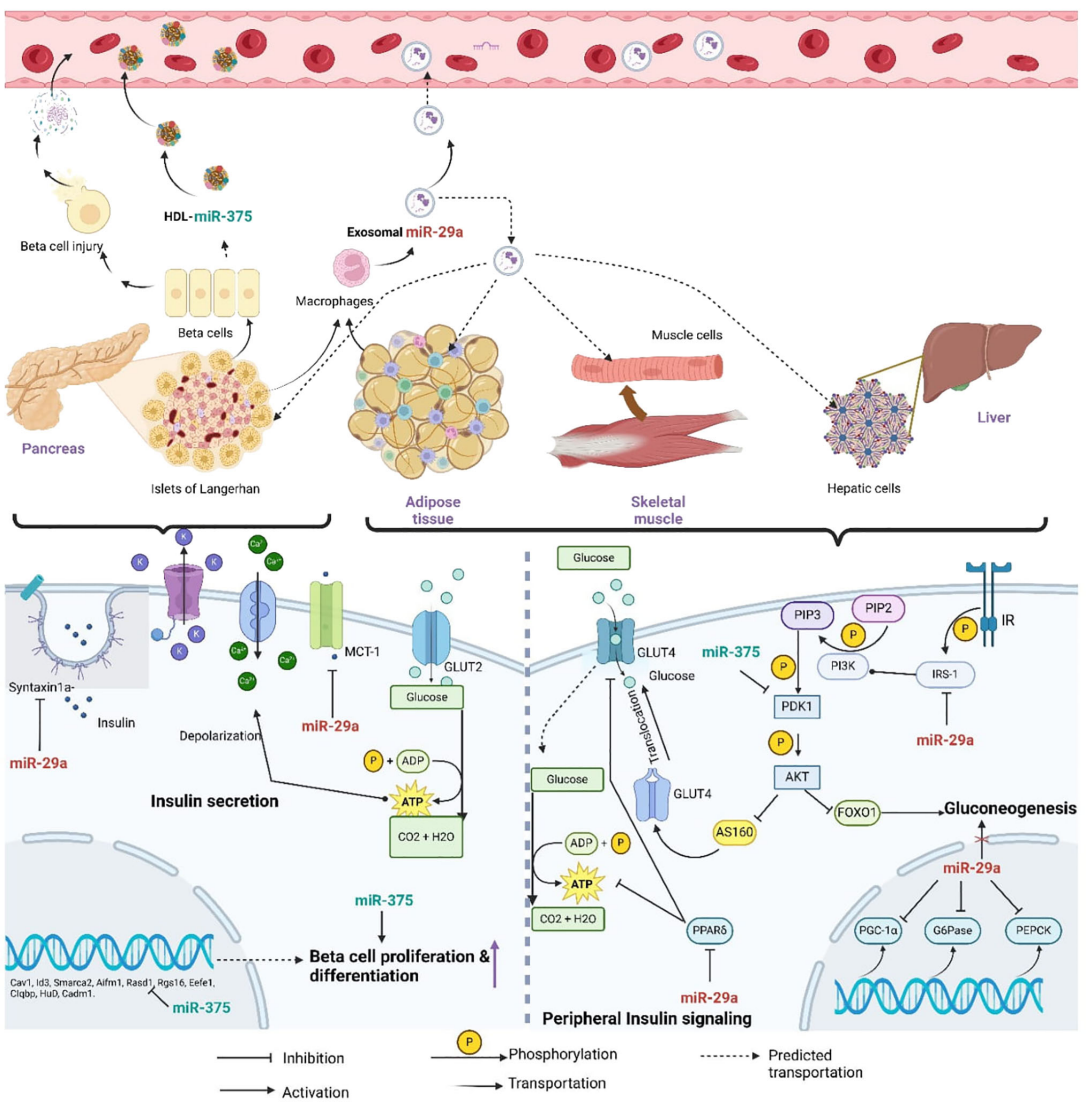
Diagram of the predicted mechanisms of horizontal transfer and the regulatory involvement of miR- 29a-3p and miR-375-3p in T2DM related molecular pathways (miR-29a, miR-29a-3p; miR-375, miR-375-3p; Syntaxin a, t-SNARE protein syntaxin -1A; MCT-1, Monocarboxylate transporter-1; GLUT2, Glucose transporter 2; GLUT4, Glucose transporter 04; ADP, Adinosine diphosphate; ATP, Adinosine triphosphate; PIP3, phosphaditylinositol triphosphate; PIP2, phosphaditylinositol bisphosphate; PI3K, phosphoinositide 3-kinase; IR, Insulin signaling receptor; IRS-1, Insulin receptor substrate; PDK1, AKT; serine/threonine kinase; PPAR, peroxisome-proliferator activated receptor δ; FOXO1, Forkhead box protein 1; PGC-1 α, peroxisome-proliferator receptor δ coactivator-1α; G6Pase, Glucose-6-phosphatase; PEPCK, Phosphoenolpyruvate carboxykinase).

According to previous studies, hsa-miR-29a-3p and hsa-miR-375-3p were up-regulated in type 2 DM plasma and involved in both beta-cell growth and function and insulin-mediated peripheral glucose uptake (9). Furthermore, *in-vitro* findings revealed that exosomal miR-29a-3p and miR-375-3p are secreted from organs associated with glucose metabolism to the circulatory system (**Figure 2**). Extracellular miRNA profiling found that plasma and serum had greater levels of hsa-miR-29a-3p. However, the expression levels of hsa-miR-375-3p varied according to the stage of type 2 DM (24). Higher expression levels were reported in the patients who were having poor glycemic control (25).

**Table 1:** Selected candidate reference miRNAs and the parameters of their optimization.

Category	miRNA (hsa-)	Forward primer	Efficiency	R^2^ value	Slope
Reference miRNA	miR-16-5p	CGCAGTAGCAGCACGTA	95.7%	0.991	-3.429
	miR-425-5p	GCAGAATGACACGATCACTC	92.10%	0.995	-3.528
	miR-22-5p	CAGAGTTCTTCAGTGGCAAG	94.80%	0.993	-3.454
	miR-191-5p	CAACGGAATCCCAAAAGC	96.2%	0.986	-3.417
Target miRNA	miR-29a-3p	CGCAGTAGCACCATCTGA	94.5%	0.980	-3.460
	miR-375-3p	AGTTTGTTCGTTCGGCTC	100.1%	0.980	-3.420

Only the primer pairs that resulted in an efficiency between 90 to 110%, R^2^ of ≥ 0.98, and a slope ≥ -3.4 were selected (28).

### In-house primer designing and validation

Primers were designed using the miRPrimer software (26). From the primer pair options provided by the software, the best-ranked pair was selected. The specificity of the selected pair was pre-evaluated using the sequence search option on the miRBase website (27). Selected primers were commercially designed (Integrated DNA Technologies (IDT), USA) and validated by producing standard curves (**Table 1**). The primer pairs and the protocol which resulted in an efficiency between 90 to 110%, R^2^ of ≥ 0.98, and a slope ≥ -3.4 were selected for further analysis (28).

**Table 2:** Demographic characteristics of participants.

Characteristics	Normoglycemic individuals	T2DM patients	p-value
N	38	53	
Age (years)	58.13 ± 9.8	57.89 ± 8.24	0.898^a^
Gender (Male/Female)	17/21	16/37	0.187^b^
HbA1c level (%)	5.26± 0.3	9.88 ± 3.07	0.000^c^*

^a^Independent T-test analysis to compare means-age ^b^Chi-Square test analysis to compare frequencies (Gender) ^c^Mann-Whitney test analysis to compare means-HbA1c level. Data of age and HbA1c level are shown as mean absorbance standard deviation (SD) as indicated. The Asterix mark (*) represents statistically significant differences.

### Statistical analysis

Raw Cq values were obtained from the Bio-Rad CFX Maestro 2.3 software (Version 5.3.022.1030) with baseline subtracted curve fit where the threshold was set at 10.0. The duplicates obtained per sample were averaged to get the mean Ct values. Demographic data were collected into a main database. All statistical evaluations were performed using SPSS Statistics Version 22 (SPSS Inc., Chicago, IL, USA) after the normality test. Probability values that were less than 0.05 were considered statistically significant. Graphical representations were generated using GraphPad Prism 8.0.1 (2018, Boston, MA). Kolmogorov-Smirnov and Shapiro-Wilk analyses were conducted to evaluate the normality of the data (**Supplementary Table 3**). After assessing the normality of the data, subsequent statistical analyses were chosen accordingly.

### Analysis of the stability

GeNorm software, which was integrated into Bio-Rad CFX Maestro 2.3 software (Version 5.3.022.1030), and BestKeeper software (29) were employed to evaluate the stability of the miRNAs.

### Analysis of the relative expression of selected target miRNAs

Relative miRNA expression of the selected miRNAs was determined by using hsa-miR-22-5p and hsa-miR-425-5p as RMs. The normoglycemic people were used as controls. The relative miRNA expression was calculated using CFX Maestro Software (Bio-Rad, USA) using the ΔΔCq method (30). The cut-off value of the FC was adjusted to 2.

## Results

### Recruitment of type 2 DM patients and normoglycemic individuals

Fifty-three (N = 53) non-hemolyzed plasma samples from type 2 DM were chosen to assess the stability in comparison to thirty-eight (N = 38) normoglycemic non-hemolyzed plasma samples (**Figure 1**, **Supplementary Table 2**). Total RNA was isolated from all the selected non-hemolyzed plasma.

### Analysis of the statistical significance-Demographic data

Both age and gender data were normally distributed according to the Kolmogorov-Smirnov and Shapiro-Wilk analyses. The independent T-test showed no significant difference between the two groups in terms of their age (p ≥ 0.05). Chi-square (2 x 2) analysis showed that there was no significant association between the two samples in terms of gender (p≥0.05).

However, HbA1c data were not normally distributed (**Supplementary Table 3**). Thus, the Mann- Whitney test was conducted to evaluate the significance of the mean difference between type 2 DM patients and normoglycemic individuals. HbA1c levels of normoglycemic individuals were significantly lower compared to type 2 DM patients (**Table 2**, p ≤ 0.05).

### Reference microRNA stability analysis of Type 2 DM patients compared to normoglycemic individuals

#### Analysis of statistical significance- quantitative PCR data

The independent t-test revealed no significant differences in baseline miRNA expression between type 2 DM patients and normoglycemic individuals for hsa-miR-425-5p, and hsa-miR-22-5p (p≥0.05, **Table 3**). The mean differences for hsa-miR-16-5p and hsa-miR-191-5p were significant (p ≤ 0.05, **Table 3**).

### Reference microRNA stability analysis by Bestkeeper and GeNorm

Between type 2 DM patients and normoglycemic controls, the mean expressions of hsa-miR-16-5p and hsa-miR-191-5p expression varied significantly (**Table 3**). According to BestKeeper analysis, the SD values of the remaining hsa-miR-425-5p and hsa-miR-22-5p were 1.10 and 1.13 respectively (**Table 3**). Both miRNAs have a CV% of less than 10%. Additionally, **Table 4** shows they had Correlation coefficient (r) values between 0.7 and 0.9. These r values were also significant (p ≤ 0.05). And power (x-fold) data of hsa-miR-425-5p and hsa-miR-22-5p were 1.55 and 1.59 respectively. According to GeNorm analysis, the hsa-miR-22-5p and hsa-miR-425-5p showed an average M value of 0.421. The miRNA with the lowest GM was hsa-miR-425-5p (GM=25.68), followed by hsa-miR-22-5p (GM = 29.85).

**Table 3:** Statistical distribution of Cq values in study participants.

Candidate miRNAs	Groups		p-value
	Normoglycemic	T2DM patients	
hsa-miR-16-5p	23.80 ± 1.39	24.62 ± 1.38	0.010*
hsa-miR-425-5p	25.58 ± 1.52	25.82 ± 1.73	0.507
has-miR-191-5p	25.70 ± 1.58	26.35 ± 1.41	0.048*
hsa-miR-22-5p	29.55 ± 1.82	30.18 ± 1.55	0.099
hsa-miR-29a-3p	30.58 ± 1.79	29.49 ± 2.16	0.020*
hsa-miR-375-3p	32.43 ± 1.33	31.22 ± 1.57	0.001*

Data are shown as mean absorbance standard deviation (SD) as indicated. Equal variance was assumed; in Levene’s test (p≥0.05). Asterix (*) represents the statistical significance.

**Table 4:** Analysis of candidate reference miRNAs by BestKeeper application software.

A. Data of housekeeping miRNAs by Best Keeper		
	hsa-miR-22-5p	hsa-miR-425-5p
N	89	89
GM	29.91	25.68
AM	29.94	25.72
min	27.36	23.14
Max	32.29	28.32
SD [+/-]	1.14	1.10
CV [%]	3.80	4.28
Power (X-fold)	1.56	1.55
	hsa-miR-22-5p	hsa-miR-425-5p
BestKeeper vs. coefficient of correlation (R)	0.703	0.725
p-value	0.001*	0.001*

GM, Geometric mean; AM, Arithmetic mean; SD, Standard deviation; CV, Coefficient of variance, Asterix (*) represents the statistically significant correlations.

### Relative miRNA expression analysis of hsa-miR-29a-3p and hsa-miR-375-3p in Sri Lankan type 2 DM patients

#### Analysis of statistical significance- quantitative PCR data

Independent t-tests revealed significant changes in baseline miRNA expression between type 2 DM patients and normoglycemic individuals for hsa-miR-29a-3p and hsa-miR-375-3p (**Table 3**).

### Relative expression analysis of selected target miRNAs

**Figure 3:**
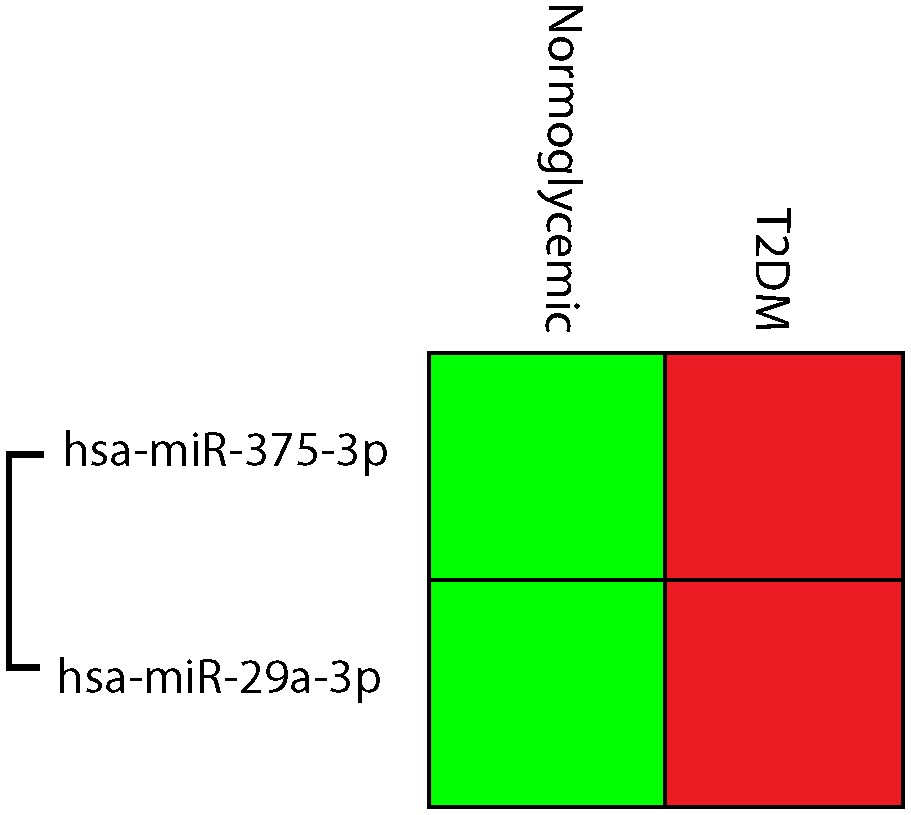
Cluster gram of normalized expressions; hsa-miR-375-3p, and hsa-miR-29a-3p, Relative higher normalized expression was indicated in red, while low normalized expressions were green.

**Figure 4:**
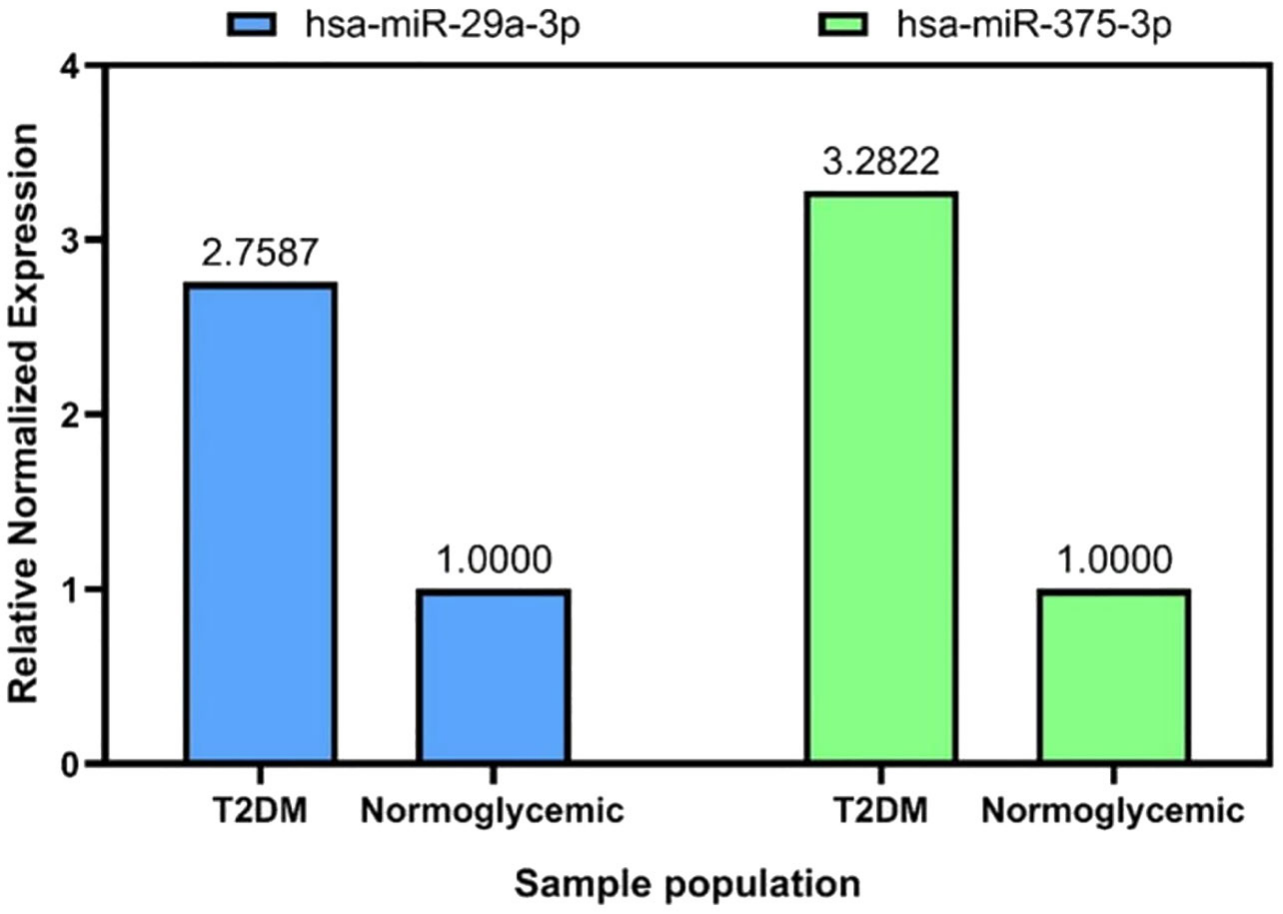
Relative miRNA expression of has-29a-3p, and has-miR-375-3p compared to normoglycemic individuals.

Type 2 DM patients had higher levels of hsa-miR-375-3p and hsa-miR-29a-3p than those who did not have diabetes (**Figure 3**). Hsa-miR-29a-3p and hsa-miR-375-3p were significantly upregulated with FCs of 2.76 and 3.28 respectively in type 2 DM patients compared to normoglycemic individuals (**Figure 4**).

## Discussion

### Hsa-miR-425-5p and hsa-miR-22-5p were stable in Sri Lankan type 2 DM patients and normoglycemic individuals

Among hsa-miR-16-5p, hsa-miR-425-5p, hsa-miR-191-5p, and hsa-miR-22a-5p, the expression levels of hsa-miR-191-5p and hsa-miR-16-5p showed statistically significant variation between the two groups (**Table 3**, p ≤ 0.05). However, a stable RM should have consistent expression levels across all samples in an experiment regardless of the disease status, i.e., normoglycemic or type 2 DM (31). Therefore, we excluded the hsa-miR-16-5p and hsa-miR-191-5p from further analysis.

Consistent with our findings, the instability of hsa-miR-191-5p and hsa-miR-16-5p was confirmed by recent investigations. The most recent investigation done by Ramanjayaneya and colleagues showed that hsa-miR-191-5p was downregulated in the plasma of type 2 DM patients once hypoglycemia was induced. Thus, plasma hsa-miR-191-5p was related to glucose metabolism (32). Another recent study showed the potential role of miR-191 and arsenic-induced mice hepatic insulin resistance. This *in-vivo* experiment conducted on livers of C57BL/6J mice exhibited that miR-191 inhibited the IRS1/AKT pathway and GLUT4 translocation (33).

Furthermore, recent studies showed significant FC in the expressions of circulating hsa-miR-16-5p in type 2 DM patients compared to non-diabetic individuals. Most recently, a miRNA profile analysis conducted on Indian type 2 DM patients showed a 3.3-fold downregulation of hsa-miR-16-5p compared to non-diabetic control (34). A study between euglycemic people, patients with impaired fasting glucose and recently diagnosed to have type 2 DM revealed similar variance in serum hsa-miR-16-5p expressions (35). Another computational analysis conducted in 2023 identified hsa-miR-16-5p as one of the dysregulated circulating miRNAs in type 2 DM (36). Even though the pre-clinical studies, including animal models and computer analysis, provide similar findings, this community-based clinical trial offered a more direct and relevant evaluation regarding the instability of hsa-miR-191-5p and hsa-miR-16-5p further strengthening the results.

To evaluate the stability of the remaining RMs, BestKeeper and Genorm algorithms were employed. Both algorithms recommended that the hsa-miR-425-5p and hsa-miR-22-5p were stable. MiRNAs that had smaller SDs, CVs less than 10%, r values close to 1, and power (x-fold) values less than 2 were generally regarded as stable in BestKeeper (29). Both miRNAs fell into the recommended cut-off values of each BestKeeper matrix except SD values. The investigated miRNAs had marginally increased SDs than the recommended value of 1 (37). Since the resulting SDs were close to 1 and other matrices (CV, r, and Power) provided acceptable values, further evaluation was conducted with GeNorm analysis. GeNorm provides a stability value (M) for each miRNA. Generally, if the M ≤ 1.5, the corresponding miRNA is considered stable (38). GeNorm identified both hsa-miR-425-5p and hsa-miR-22-5p miRNAs were under the acceptable range to consider them stable.

Unpresidently, our study found that hsa-miR-425-5p and hsa-miR-22-5p were stable in type 2 DM patients and normoglycemic individuals. However, a recent publication by Liu et al. reported that serum hsa-miR-425-5p level exhibited a gradual increment in the healthy control group, DM patients without DR, and DR patients (39). Therefore, they have suggested hsa-miR-425-5p as the potential prognostic miRNA for DR (39). Interestingly, the miRNA expression changes were minimal between healthy controls and DM without DR, but greater between healthy controls and those with DR. Importantly, our study evaluated the stability between normoglycemic vs type 2 DM. Furthermore, this discrepancy could be due to the reference (U6) which was used for normalization in Liu’s experiment. U6 was a non-coding RNA that was suggested to be unstable among type 2 DM patients and patients with DR (31). Nonetheless, the comparatively larger sample size in our study offered more reliability and validity for our findings.

However, these contrasting findings warrant further research to elucidate the plasma expression of hsa-miR-425-5p in type 2 DM against normoglycemic individuals. In addition, no prior research was found that examined the plasma expression levels of hsa-miR-22-5p in type 2 DM patients.

### Hsa-miR-29a-3p and hsa-miR-375-3p were highly expressed in Sri Lankan type 2 DM patients

Hsa-miR-29a-3p and hsa-miR-375-5p were two main miRNAs that were differentially expressed in type 2 DM populations. Both miRNAs were elevated in Sri Lankan type 2 DM patients compared to normoglycemic individuals when normalized using stable miRNAs hsa-miR-425-5p and hsa-miR-22-5p.

Previous studies on plasma and serum revealed an elevated level of hsa-miR-29a-3p, which was consistent with our findings. According to a meta-analysis published in 2015, miR-29a was the most often reported and elevated miRNA in the circulation of all examined miRNA profiling studies on type 2 DM patients (N=10) (9). MiR-29a-3p was shown to be consistently elevated in type 2 DM patients and even highly expressed in prediabetic people (40). Overweight women tend to have decreased levels of miR-29a-3p, if treated with metformin to improve their hepatic insulin sensitivity (41). In type 2 DM patients, serum miR-29a-3p levels were considerably greater than in healthy persons whereas the relative concentration of miR-29a was determined using miR2911 as a reference. The average relative concentration of miR-29a in healthy people was 11.30 nmol/L, whereas in type 2 DM patients it was 20.66 nmol/L (42). Obese women with early gestational DM (GDM) had higher miR-29a-3p levels, indicating a direct relationship between miR-29a-3p overexpression and impaired glucose metabolism (43).

Importantly, the suggested regulatory role of miR-29a-3p was in line with the observed expression patterns. As elaborated in **Figure 2**, intercellularly elevated miR-29a-3p relates to pancreatic beta-cell insulin secretion, beta-cell inflammation, and insulin signaling pathways (44–47). Further, it has been postulated that exosomal miR-29a could be produced by macrophages associated with adipose tissues (48). Exosomes secreted by macrophages present in the adipose tissues were found to have a higher amount of miR-29a. This exosomal miR-29a transfers into nearby adipocytes, myocytes, and hepatocytes to induce insulin resistance (49).

On the other hand, previous findings on the plasma expression level of hsa-miR-375-3p in type 2 DM patients were less conclusive. Some studies found that miR-375 was increased in type 2 DM patients compared to normoglycemic persons, while others found the opposite or no significant difference. Zhu et al. revealed that it was the most highly elevated miRNA in examined human profiling investigations (9). Similar findings were published in 2014, where serum concentrations of miR-375 were five times greater in type 2 DM patients than in individuals with normal glucose tolerance (50). Significant upregulation of hsa-miR-375-3p was found in the serum of newly diagnosed type 2 DM patients compared to normoglycemic individuals who were susceptible to type 2 DM. Interestingly, in that study, the expression level of hsa-miR-375 was similar in individuals with impaired fasting glucose and normoglycemic individuals (51). However, overexpression of miR-375-3p in the type 2 DM circulatory system was confirmed by *in vitro* and *in vivo* investigations (24).

In contrast, a clinical study indicated that, when compared to patients with normal glucose tolerance, plasma expressions of miR-375 were downregulated in patients with impaired glucose tolerance but raised in patients with type 2 DM (25). Similarly, a recent investigation suggested that hsa-miR-375-3p was significantly downregulated in the plasma of newly diagnosed type 2 DM patients, and type 2 DM susceptible subjects compared to healthy controls (52). However, the underlying explanation for this discrepancy has yet to be resolved.

MiR-375-3p promotes beta-cell growth and function (24). When beta-cells were stressed or died, miR-375-3p was released into the bloodstream. As a result, greater levels of miR-375-3p might serve as a marker for beta-cell damage (**Figure 2**). According to the literature, beta-cell damage in type 2 DM occurred gradually, beginning in the early stages (53, 54). Therefore, we may assume that the miR-375-3p expression may depend on which stage the studied population was in throughout the experiment. However, the higher expression level of hsa-miR-375-3p in our work was highly supported by the proposed regulatory role of miR-375-3p in type 2 DM. MiR-375 has been vastly experimented with for its regulatory role in beta-cell proliferation due to its high expression in human pancreatic islet development (24, 55) (**Figure 2**).

However, methodological differences could be one of the causes for the inconsistent findings related to miR-375. Previous studies that evaluated plasma had not reported their hemolysis status although the miR-375 level in plasma was extremely vulnerable to hemolysis (25, 52). In contrast, only the non-hemolyzed plasma samples were evaluated in our experiment. Validated RMs were utilized to explore the relative expression. Furthermore, our tested type 2 DM patients had poor glycemic control (Mean HbA1c level = 9.88+/-3.07). Furthermore, the higher expression level of hsa-miR-375-3p in our work was highly supported by the proposed regulatory role of miR-375-3p in type 2 DM. Furthermore, the results of the relative expression depend on the reference control used (56). Some of the RMs employed in the stated studies were not verified and demonstrated to have a regulatory effect on type 2 DM, i.e., miR-191-5p (32, 52). For hsa-miR-375-5p, despite prior inconsistencies, our improved methodology revealed upregulation, aligning with its proposed role in type 2 DM development.

While this study paved the way for future investigations offering promising results, it is important to acknowledge the limitations of the study. One of the limitations was the hemolysis that restricted the sample sizes. Mostly improper venipuncture and forceful aspiration of blood would cause hemolysis (57). However, given the involvement of trained nurses in blood collection, observed hemolysis due to improper technique was unlikely, yet could not be entirely excluded. The storage time of plasma and mechanical disruption due to transportation would cause hemolysis (58). However, compared to previous validation studies, we managed to achieve a comparable sample size in terms of patients and controls (**Supplementary Table 1**) (31, 59, 60). During this experiment, DNase treatment was not conducted for the extracted RNA. The serum/plasma contains very low levels of DNA. Furthermore, the extracted RNA contains very low levels of miRNAs. Thus, the DNase treatment could reduce the miRNA yield (61). The convenient sampling method that was used for selecting participants with normal blood sugar levels would limit the generalizability of the findings to the entire normoglycemic of Sri Lanka. Thus, more related investigations for broader representative samples are recommended in the future to establish the validity of these RMs further to the Sri Lankan type 2 DM population.

## Conclusion

To conclude, the human plasma expressions of hsa-miR-425-5p and hsa-miR-22-5p were stable in both type 2 DM patients and normoglycemic individuals in Sri Lanka. This study represents a novel contribution to the understanding of stable miRNAs in type 2 DM patients. While the lack of research underscores the need for further exploration, this study on the stability of hsa-miR-425-5p and hsa-miR-22-5p offers valuable groundwork for future investigations about stable miRNAs in type 2 DM plasma. Higher expression of hsa-miR-29a-3p and hsa-miR-375-3p was present in type 2 DM patients compared to normoglycemic individuals in Sri Lanka. Future studies will have to apply replications with balanced and matched cohorts to verify the identified miRNAs as T2DM diagnostic markers. Logistic regression and Area under the curve analysis should be employed in the inclusion to quantify diagnostic performance and compare against standard blood glucose assays. Supplemental analysis has to be applied to strengthen discoveries and increase the clinical application of the identified miRNAs.

## Data Availability

The original contributions presented in the study are included in the article/**Supplementary Material**, further inquiries can be directed to the corresponding author/s.

## Glossary

3’-UTR: 3 ends untranslated region
5’UTR: 5 end untranslated region
AD: Alzheimer’s Disease
ADA: American Diabetes Association
ADP: Adenosine diphosphate
AKT: Serine/threonine kinase
AM: Arithmetic mean
ATP: Adenosine triphosphate
cDNA: Complementary DNA
Cq: Quantitative cycles
CV: Coefficient of variance
DLBCL: Diffuse Large B-cell lymphoma
DM: Diabetes mellitus
FOXO1: Forkhead box protein 1
G6Pase: Glucose-6-phosphatase
GLUT2: Glucose transporter 2
GLUT4: Glucose transporter 4
GM: Geometric mean
HbA1c: Glycated hemoglobin 1c
HF: Heart failure
hsa-: Homo sapiens
IDT: Integrated DNA Technologies
IFCC: International Federation of Clinical Chemistry
INSR: Insulin signaling receptor
IRS-1: Insulin receptor substrate
IS: Insulin signaling
LTBI: Latent Tuberculosis infection
MCI: Mild Cognitive Impairment
MCT-1: Monocarboxylate transporter-1
miRNA: microRNA
mRNA: messenger RNA
NC-RNA: Non-coding RNA
NCLC: Non-small cell lung carcinoma
NGSP: National Glycohemoglobin Program
NHSL: National Hospital of Sri Lanka
PD: Pyruvate dehydrogenase kinase 1
PEPCK: Phosphoenolpyruvate carboxykinase
PI3K: Phosphoinositide 3-kinase
PIP2: Phosphatidylinositol bisphosphate
PIP3: Phosphatidylinositol triphosphate
PPAR: Peroxisome proliferator-activated receptor δ
PGC-1ὰ: Peroxisome proliferator receptor δ coactivator-1α
RG: Reference gene
RM: Reference microRNA
RT-qPCR: Real-time quantitative polymerase chain reaction
SD: Standard deviation
Syntaxin 1a: t-SNARE protein syntaxin-1A
TB: Tuberculosis
VC: Vulvar squamous carcinoma
VINL: Vulvar intraepithelial neoplasia lesions
WHO: World Health Organization
