# Supplementary material for: Identification of stable reference genes and differential miRNA expression in Sri Lankan type 2 diabetes mellitus patients: a cross-sectional study
Source: Front Endocrinol (Lausanne). 2025 Jun 12;16:1554827. doi: 10.3389/fendo.2025.1554827 (PMC12197912; doi:10.3389/fendo.2025.1554827)
Supplement: Supplementary file 1 [file DataSheet1.pdf]

Supplement 01 Previous stability validation studies and miRNA expression studies that have used the selected candidate miRNAs as reference controls.

Reference microRNAs	Validation study (VS)/Used as endogenous control (EC)	Disease	Biofluid/organ tested	Sample	Reference
miR-16-5p	VS	Osteogenesis imperfecta	Serum	Osteogenesis imperfecta patients (N=16). Healthy controls L (N=08)	(Wang *et al.*, 2012)
	VS	Cardiovascular disease	Serum	HF patients (N=25). Hypertension patients (N=10), Healthy control (N=27)	(Wang *et al.*, 2018)
	EC	Breast Cancer	Plasma	Early-stage breast cancer patients (N=20), Healthy control (N=20)	(Zhao *et al.*, 2010)
	EC & VS	Metastatic breast cancer	Serum	Primary breast cancer patients (N=59), Metastatic disease patients (N=30), Healthy control (N=29)	(Roth *et al.*, 2010)
	EC	B-Cell lymphoma	Serum	DLBCL patients (N=60), Healthy control (N=43)	(Lawrie *et al.*, 2008)
	VS	Non-small cell lung carcinoma	Plasma	NCLC Patients (N=30), Healthy individuals (N=30)	(Sourvinou, Markou and Lianidou, 2013)

miR-16-5p &miR-425-5p	VS	Breast Cancer	Whole Blood	Newly diagnosed breast cancer patients (N=50), Healthy control (N=30)	(McDermott, Kerin and Miller, 2013)
miR-425-5p	VS	Vulvar intraepithelial neoplasia lesions and vulvar squamous carcinoma	Plasma	VINL Patients (N=17), VC patients (N=27)	(Zalewski *et al.*, 2017)
	VS	Tuberculosis	Plasma	Pulmonary TB patients (N=12), LTBI patients (N=12), Healthy control (N=12)	(Barry *et al.*, 2015)
miR-191-5p	VS	Breast cancer	Serum	Breast cancer patients (N=48), Healthy control (N=48)	(Hu *et al.*, 2012)
	VS	Colorectal Adenocarcinoma	Serum	Colorectal Adenocarcinoma patients (N=30), Colorectal Adenoma patients (N=25), Healthy Control (N=30)	(Zheng *et al.*, 2013)
miR-22-5p	VS	Osteoarthritis	EVs from cartilage, adipose tissue, and Bone Marrow cells	Osteoarthritic patients (N=03)	(Ragni *et al.*, 2021)
	VS	Osteoarthritis	EVs of Adipose tissue-derived Mesenchymal Stem Cells	Osteoarthritis patients (N=05)	(Ragni *et al.*, 2019)

	VS	Alzheimer's disease	Plasma	Subjects without AD (N=24), Subjects with MCI (-) (N=22), Subjects with MCI (+) (N= 22)	(Dakterzada *et al.*, 2020)

HF: Heart Failure, DLBCL: Diffuse Large B-cell lymphoma, NCLC: Non-small cell lung carcinoma, VINL: Vulvar intraepithelial neoplasia lesions, VC: Vulvar squamous carcinoma, TB: Tuberculosis, LTBI: Latent Tuberculosis infection, AD: Alzheimer's Disease, MCI: Mild Cognitive Impairment.
