# Supplementary material for: Identification of stable reference genes and differential miRNA expression in Sri Lankan type 2 diabetes mellitus patients: a cross-sectional study
Source: Front Endocrinol (Lausanne). 2025 Jun 12;16:1554827. doi: 10.3389/fendo.2025.1554827 (PMC12197912; doi:10.3389/fendo.2025.1554827)
Supplement: Supplementary file 2 [file DataSheet2.pdf]

Supplement 02 Nanodrop spectral analysis results of selected plasma samples.

Sample	Mean Spectral absorbance at 414 nm
Type 2 diabetes mellitus patients (N=53)	0.161±0.09
Normoglycemic individuals (N=38)	0.114±0.02

Data are shown as mean absorbance ± standard deviation (SD) as indicated.
