# Supplementary material for: Identification of stable reference genes and differential miRNA expression in Sri Lankan type 2 diabetes mellitus patients: a cross-sectional study
Source: Front Endocrinol (Lausanne). 2025 Jun 12;16:1554827. doi: 10.3389/fendo.2025.1554827 (PMC12197912; doi:10.3389/fendo.2025.1554827)
Supplement: Supplementary file 3 [file DataSheet3.pdf]

Supplement 03 Kolmogorov-Smirnov and Shapiro-Wilk analysis results for normality of the data set

		**Population**	**Kolmogorov-Smirnov (p value)**	**Shapiro-Wilk (p value)**
qPCR data	hsa-miR-16-5p	T2DM patients	0.173	0.601
		Normoglycemic	0.200	0.248
	hsa-miR-425-5p	T2DM patients	0.200	0.497
		Normoglycemic	0.200	0.577
	hsa-miR-191-5p	T2DM patients	0.200	0.265
		Normoglycemic	0.200	0.475
	hsa-miR-22-5p	T2DM patients	0.096	0.052
		Normoglycemic	0.200	0.328
	hsa-miR-29a-3p	T2DM patients	0.200	0.434
		Normoglycemic	0.063	0.076
	hsa-miR-375-3p	T2DM patients	0.095	0.061
		Normoglycemic	0.134	0.070
Age		T2DM patients	0.200	0.886
		Normoglycemic	0.200	0.906
HbA1c level		T2DM patients	0.014*	0.000*
		Normoglycemic	0.010*	0.008*

If the data were normally distributed, p-value≥ 0.05. The Asterix mark (*) represents the significant deviations from normal distribution.
